# 2,2′-Di­methyl-7,7′-(methyl­enedi­imino)di-1,8-naphthyridin-1-ium bis(perchlorate). Erratum

**DOI:** 10.1107/S2056989019015937

**Published:** 2020-02-28

**Authors:** IUCr Editorial Office

**Affiliations:** a 5 Abbey Square, Chester CH1 2HU, England

## Abstract

This article reports the correction of a paper by Mo *et al.* [*Acta Cryst.* (2008), E**64**, o1702].

## Abstract 2

Erratum to *Acta Cryst.* (2008), E**64**, o1702.

After thorough investigation, an article by Mo *et al.* (2008 ▸) has been shown to have problems with its data set. The article is therefore withdrawn from the published literature.

## References

[bb1] Mo, J., Liu, J.-H., Pan, Y.-S., Zhang, S.-M. & Du, X.-D. (2008). *Acta Cryst.* E**64**, o1702. 10.1107/S1600536808024616 PMC2960735 21201691

